# Nurses' contribution to organizational health literacy: a conceptual analysis informed by a focused mini-review

**DOI:** 10.3389/fpubh.2026.1856603

**Published:** 2026-09-23

**Authors:** Graeme Drummond Smith, Ann Bonner, Thi Thuy Ha Dinh, Hemamali Jagodage

**Affiliations:** 1 S. K. Yee School of Health Sciences, Saint Francis University, Hong Kong, Hong Kong SAR, China; 2 School of Nursing and Midwifery, Griffith University, Nathan, QLD, Australia; 3 Monash University, Melbourne, VIC, Australia

**Keywords:** communication, health literacy, healthcare, nursing, organizational health literacy, social determinants of health

## Abstract

Organizational health literacy (OHL) shifts attention from individuals' health literacy (HL) skills to the ways healthcare organizations make information, services and care pathways accessible, understandable and usable. Nurses are central to health-related communication, education, care coordination and quality improvement, yet their contribution to OHL remains under-documented. This conceptual article develops a nursing-informed perspective on OHL, supported by a focused mini-review of recent studies. The review was used to identify illustrative examples of how OHL has been examined in nursing-relevant contexts, rather than to provide a comprehensive evidence synthesis. We argue that nurses may contribute to OHL by supporting clear communication, care navigation, shared decision-making, co-design of information resources, quality improvement and implementation of HL-sensitive systems. Mapping Nutbeam's functional, interactive and critical levels of HL to organizational responsibilities highlights how nursing practice can contribute not only to individual understanding, but also to system-level responsiveness and equity. Strengthening nurse-led OHL will require leadership support, workforce development, use of assessment tools, consumer partnership, and integration into quality, safety and accreditation structures.

## Introduction

Health literacy (HL) is a multidimensional construct that encompasses the knowledge, skills, and motivation required for people to access, understand, appraise, and apply health information in ways that support health across the life course. It is shaped by a range of determinants, including cultural and social norms, the structure and responsiveness of healthcare systems, and personal and situational factors (2). The World Health Organization (WHO) has recently defined HL as “representing the personal knowledge and competencies that accumulate through daily activities, social interactions and across generations,” with these personal competencies being mediated by the demands and complexity of the health system and the broader social context. This definition underscores that HL is not solely an individual attribute but is co-produced in interaction with health services, inclusive of healthcare providers, and society (3). Adequate HL can contribute to improving health outcomes and reducing avoidable and unjust health inequities across populations. Additionally, the Office of Disease Prevention and Health Promotion (4) positioned HL as a foundational principle and overarching goal for improving population health and wellbeing. This positioning explicitly recognizes that responsibility for HL rests not merely with individuals, but also with healthcare organizations and systems, which must provide health-related information in an equitable, accessible, and person-centered ways (4). In healthcare settings, lower levels of HL have been associated with higher mortality, greater morbidity, and increased rates of hospitalization (5).

## Health literacy

In seminal work, Nutbeam (6) proposed three progressive levels of HL: (i) functional HL, involving the basic reading and writing skills required to understand factual health-related information in everyday health contexts; (ii) interactive/communicative HL, which involves more advanced cognitive and social skills that allow individuals to actively participate in communication, extract health information from different sources, and apply it to personal circumstances; and (iii) critical HL, which embraces higher-level cognitive and social skills needed to critically evaluate health-related information, understand broader determinants of health, and use this understanding to exercise control over health decisions and engage in collective action to improve health and equity (6, 7).

Although it was originally developed to describe individual HL, Nutbeam's three-level typology can be meaningfully mapped onto OHL by considering how organizations support or hinder each level. At the functional level, health-literate organizations provide clear, readable, and navigable information and services to enable users to understand and use basic information. At the interactive level, the healthcare providers in these organizations create opportunities for two-way communication and shared decision-making, train staff in communication skills, and encourage questions. These practices ensure that service users can actively engage, clarify information, and apply it to their own circumstances. At the critical level, healthcare organizations support people and communities to critically appraise information, recognize structural determinants of health, participate in co-design and governance, and advocate for changes that improve health and equity, extending Nutbeam's critical level of HL from individuals to collective and system-level action (8). Recognizing these three levels of HL can inform the design of health-literate organizations, especially when focusing on how organizational structures and communication either constrain or enable people's functional, interactive, and critical engagement with health information and services (9).

## Frameworks of organizational health literacy

In 2012, the Institute of Medicine Roundtable on HL proposed the “Ten Attributes of Health Literate Health Care Organizations” which describes how organizations can embed HL into leadership, governance, and quality improvement; prepare and support their workforce to communicate clearly; and actively involve service users in designing and evaluating information and services (10, 11). However, evidence for its effectiveness as an implementation guide is limited, due to few robust evaluations of impacts on patient outcomes, equity, or system performance. As the framework was developed in high-income healthcare systems and pays limited attention to social determinants of health and contextual adaptation, there is a need for more flexible and context-sensitive OHL frameworks (12).

In response, several other OHL frameworks have been developed. The Vienna Concept of Health-Literate Hospitals and the associated V-HLO self-assessment tool operationalize OHL as a set of elements and indicators for healthcare organizations, particularly in hospital settings (13). The Organizational Health Literacy Responsiveness (Org-HLR) framework further highlights leadership, organizational culture, workforce, communication, and community engagement as key dimensions of responsiveness (14). These emerging frameworks explicitly prioritize preventive action and intersectoral collaboration across sectors such as healthcare and education, emphasizing specific attention to social determinants and context (15).

These OHL frameworks are particularly relevant to the nursing profession because they highlight how nurses, positioned at the frontline of patient care, can enact and advocate for system-level practices that make healthcare environments more navigable and health-literate for patients and their family members. Nurses and other health professionals can use OHL self-assessment tools to reflect on current practice, rate OHL within their services, and prioritize improvements that enhance the understandability and accessibility of health-related information for those in their care (16). In parallel, a growing body of evidence describes nurses' roles in supporting HL, portraying nurses as direct care providers, facilitators, educators, and advocates who help individuals access, understand, appraise, and use health-related information across a range of healthcare settings (17–19).

In a recent review, Smith et al. (20) argued that HL should be understood not only as an individual patient trait but also as an organizational responsibility, outlining how OHL can help kidney services better support patients' ability to understand and use complex treatment information by tailoring communication and systems to patients' linguistic, cultural, educational, and socioeconomic contexts. They called for renal healthcare leaders, including nurses and multidisciplinary teams, to prioritize OHL initiatives as part of efforts to support improvements and reduce inequities. Building on this and related work, there is a need to more clearly articulate how nurses across settings can enact and lead OHL at the point of care and within organizational structures.

This paper develops a nursing-informed conceptual perspective on OHL, supported by a focused mini-review of recent empirical literature. The mini-review was used to identify illustrative examples of how OHL has been applied in nursing-relevant contexts, rather than to provide a comprehensive synthesis of all available evidence.

## Methods

A review protocol was not registered because this article was designed as a focused mini-review to inform a conceptual analysis, rather than as a systematic review. Nevertheless, the search scope, eligibility criteria, screening approach, and data-charting process are reported to support transparency. With assistance from a research librarian, the following Boolean search string was developed to combine terms for OHL and nursing:

(“organizational health literacy” OR “organizational health literacy”

OR “institutional health literacy”

OR “health literate organization^*^” OR “health literate organization^*^”

OR “health literate healthcare organization^*^” OR “health literate healthcare organization^*^”

OR “health literacy responsive organization^*^” OR “health literacy responsive organization^*^”

OR “health literacy responsiveness”)

AND nurs^*^

Searches were conducted in CINAHL Complete (EBSCOhost), British Nursing Database (ProQuest), MEDLINE (Ovid), and PubMed. PubMed contains MEDLINE-indexed citations as well as additional publisher-supplied and non-MEDLINE records; therefore, records from MEDLINE and PubMed were exported and deduplicated before screening. No setting-related search block was applied because the conceptual analysis aimed to capture nursing-relevant OHL literature across hospital, acute-care, primary-care, community, residential-care, and other healthcare settings. This approach was selected to avoid systematically excluding hospital-based OHL literature that does not use public health or community health related terminology. Studies published in English between January 2020 and August 2026 were considered, which was conducted on 31st March 2026 and rerun 1st August 2026. Studies were included if they addressed OHL in healthcare or care settings and had clear relevance to nursing practice, nursing systems, or nurse-led implementation. Studies were excluded if they focused only on individual patient health literacy without an organizational component see Figure 1.

**Figure 1:**
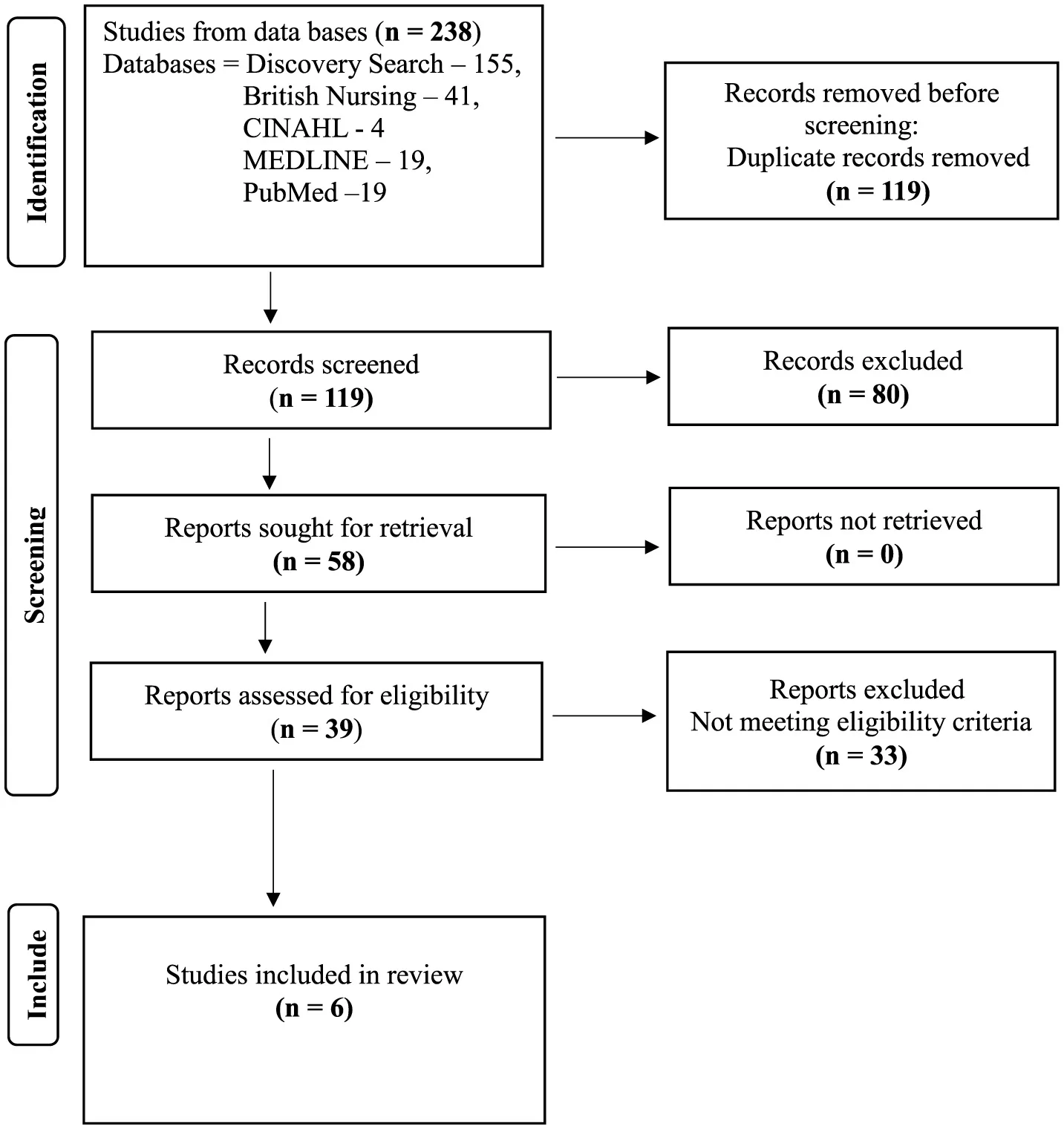
PRISMA flow diagram of included studies.

The retrieved literature was screened by the first author (^***^) to identify recent empirical studies offering illustrative nursing-relevant examples of OHL. Studies judged to be relevant were reviewed in full text, and key information was charted narratively. Extracted data were summarized using a narrative approach. The six illustrative empirical studies were organized around four pre-specified areas to inform the nursing-focused conceptual analysis of OHL: conceptualizations of OHL; use of OHL frameworks and assessment tools; organizational enablers and barriers relevant to nursing practice; and implementation strategies. One reviewer charted data from the included articles using a structured extraction form, the completed extraction for accuracy and completeness were cross-checked and any discrepancies were discussed and resolved by consensus. This process was intended to inform the conceptual analysis rather than generate a formal evidence synthesis.

### Insights from the focused mini review

The empirical studies identified to inform this conceptual analysis described OHL across a range of nursing-relevant healthcare settings. Chang et al. (21) focused on primary care, surveying community-based nurses in Taiwan. Häberle et al. (22) examined nurses in acute care by surveying managers in German hospitals. Zhu et al. (23) explored barriers to OHL in nurses in Chinese hospitals caring for older adults, while Wu et al. (24) explored health-literate nursing systems using a grounded theory design in China. Rathmann et al. (25) investigated healthcare provision, including nursing, in facilities for people with disabilities in Germany, representing long-term residential and social care contexts. Stavdal et al. (26) studied nurses in a surgical ward in a Norwegian hospital, evaluating a HL intervention for registered nurses in an inpatient setting. Another German study provided a process evaluation of an organization-wide HL intervention with strong emphasis on contextual barriers, leadership, change agents and the role of nurses (27). Straßmayr et al. (1) conducted a feasibility study of an OHL tool in European hospitals, reporting that comprehensive OHL tools can stimulate organizational reflection and consensus building, whilst also underscoring the need to tailor toolkits to different professional groups, including nursing staff see Table 1.

**Table 1:** Characteristics of included studies.

Study	Country/Setting	Study aim	Design	Participants	Key findings
Chang et al. (21)	Taiwan; community health centers	Evaluate OHL from an institutional perspective	Cross-sectional	Community health center staff, including nurses	• Nurses were primary frontline providers of health information, education, and navigation support. • Organizational policies and managerial attention to communication were identified as enabling conditions; however, variability in institutional commitment and limited structural supports constrained consistent delivery of health-literacy-sensitive nursing care across centers.
Häberle et al. (22)	Germany; acute care hospitals	Assess the level of OHL maturity from a management perspective	Cross-sectional	Hospital managers and senior leaders	• Moderate levels of organizational health literacy were reported at a strategic level. Leadership commitment and integration of OHL into quality and safety frameworks were identified as key enablers. • Nurses were positioned as central implementers of OHL in clinical practice; however, limited operationalization reduced the translation of strategic intent into supportive conditions for everyday nursing work.
Rathmann et al. (25)	Germany; facilities for people with disabilities	Explore OHL in disability care facilities	Mixed methods	Managers and staff, including nurses	• Nurses had an intermediary role in enabling access to and understanding of health information for service users with disabilities. Availability of target-group-specific information and health-literate physical and organizational environments supported nursing practice, while fragmented organizational approaches and absence of systematic OHL frameworks limited consistency.
Stavdal et al. (26)	Norway; hospital nursing workforce	Develop and feasibility-test a health literacy intervention for registered nurses	Quasi-experimental feasibility trial	Registered nurses	• Structured health-literacy training was feasible and acceptable for nurses and supported development of health-literacy-sensitive communication practices. • Findings highlighted that health literacy education remained largely *ad hoc* and insufficiently embedded within organizational professional development systems, reinforcing reliance on individual nurses rather than sustained organizational supports.
Wu et al. (24)	China; health service organizations	Develop a conceptual framework for a health-literate nursing system	Grounded theory	Nurses, nurse managers, and health system stakeholders	• Nurses were conceptualized as central actors within a health-literate system requiring alignment between leadership, organizational culture, workforce capability, and systems. • Enabling conditions included leadership engagement and coherent organizational processes, while barriers related to system fragmentation, organizational silos, and misalignment between strategic goals and nursing practice.
Zhu et al. (23)	China; hospitals caring for older adults	Explore barriers to the development of OHL from multiple perspectives	Mixed-methods	Managers, health professionals (including nurses), and older patients	• Nurses were responsible for delivering health-literacy-sensitive care to older patients with complex needs. • Substantial organizational barriers dominated, including limited leadership commitment, staffing and time constraints, inadequate health literacy training, and rigid organizational structures, with few enabling conditions identified to support nursing practice.

OHL, organizational health literacy.

Overall, the studies were conducted in diverse high-income countries using various study designs–cross-sectional (21, 22), mixed-methods (23, 25), grounded theory (24), quasi-experimental pre–post feasibility trials (1, 26) and implementation studies (27). The following areas are intended as a nursing-informed conceptual interpretation of illustrative literature, not as an exhaustive account of all evidence on OHL see Table 2.

**Table 2:** Mapping of included studies to organizational health literacy domains.

Study	Leadership and governance	Workforce capability (Nursing)	Communication and information practices	Organizational systems and environment
Chang et al. (21)	✓	✓	✓	✓
Häberle et al. (22)	✓	*o*	✓	✓
Rathmann et al. (25)	✓	✓	✓	✓
Stavdal et al. (26)	*o*	✓	✓	*o*
Wu et al. (24)	✓	✓	✓	✓
Zhu et al. (23)	✓	✓	✓	✓

✓, Requirement met; *o*, Requirement not met.

#### Conceptual area 1: from individual health literacy to organizational responsibility

The reviewed work reconceptualizes HL from an individual patient attribute to an organizational responsibility, emphasizing that nurses working in hospitals and community services must ensure information and care processes are accessible, understandable, and usable for diverse populations (21). Building on this reconceptualization, nurses are positioned as key intermediaries of OHL, translating institutional commitments into concrete communicative, environmental, and procedural adaptations at the point of care. By tailoring explanations, revising written and visual materials, and working closely with multidisciplinary teams, nurses can actively co-produce HL-responsive systems that mitigate literacy-related barriers experienced by individuals, rather than expecting individuals and families to compensate for organizational complexity (23). Healthcare organizations are increasingly portrayed as having a responsibility to design systems, pathways, and environments that reduce literacy-related barriers, rather than expecting patients and families to compensate for complex and jargon-laden communication. Straßmayr et al. (1) exemplified the shift from individual HL toward OHL by using a hospital-level self-assessment tool (OHL-Hos) to evaluate and develop structures, processes, and policies that strive to make care more health literate. Zhu et al. (23) highlighted how older adults in the hospital settings can face multiple intersecting vulnerabilities, such as sensory impairments, cognitive decline, and limited formal education, which heighten the importance of organizational responsibility for HL-sensitive care. Furthermore, they reported that nurses describe the need to adjust communication, redesign information materials, and coordinate with other disciplines to make institutional processes more navigable for older patients and their caregivers (23). Similarly, Rathmann et al. (25) suggested that in facilities for people with disabilities, staff, including nurses and healthcare support workers, must adapt communication, routines, and the physical environment to support residents' understanding and participation in care decisions. Finally, Lubasch et al. (27) framed “professional health literacy” and “health literate hospitals” as organizational qualities built through systems, training, and infrastructure, supporting a shift from individual HL to OHL as a core responsibility. Collectively, these studies suggest that nurses increasingly understand HL as something that is co-produced in interaction with organizational structures and processes, rather than as a deficit located within individuals.

#### Conceptual area 2: OHL frameworks and assessment tools as levers for nursing-led improvement

OHL frameworks and self-assessment tools, such as the International Self-Assessment Tool for Organizational Health Literacy in Hospitals (OHL-Hos) and the Health Literate Health Care Organization 10-item questionnaire (HLHO-10), translate abstract OHL standards into concrete indicators that hospitals and other healthcare settings can use to appraise organizational practices. Within OHL frameworks, nurses occupy a potentially important position in interpreting assessment findings and translating them into practice-level change by using tools such as OHL-Hos, Org-HLR, and the HLHO-10. These tools can assist nurses with identifying gaps in communication, navigation support, and staff education. They can also support nurses to lead or co-lead the participatory development and review of health information materials, ensuring that organizational responses are understandable, accessible, and responsive to the needs of people with disabilities and other vulnerable groups (25). Specifically, Rathmann et al. (25) used the HLHO-10 in rating readiness of facilities for people with disabilities in Germany and illustrated below-average OHL scores among healthcare managers, particularly in relation to the participative development of health information. Related OHL self-assessment tools, including OHL-Hos and the Organizational Health Literacy Responsiveness (Org-HLR) tool, can be used to assess domains such as leadership, communication, navigation, patient involvement, and staff training, providing a structured lens through which nurses and other staff can examine their organizations' health-literacy responsiveness. Also in Germany, HL-COM was used in hospital settings to link communication quality to patient experiences and unmet information needs, illustrating the practical value of OHL measurement for nursing professionals (27). At the level of individual clinicians, Stavdal et al. (26) measured the health-literacy sensitivity of registered nurses using the Instrument of HL Competencies, discussing gaps in nurses' knowledge, skills, and attitudes for meeting the HL needs of patients in their care. Although the Instrument of HL Competencies assesses individual professional competencies rather than OHL, workforce capability constitutes a central domain of major OHL frameworks, including the Vienna Concept and Org-HLR, supporting its relevance. This study's intervention showed that nurses' HL competencies can be enhanced through targeted education, but also highlighted that sustained change requires alignment with organizational policies and support (26). Wu et al. (24) conceptualized how OHL-oriented tools may support clinical nursing practice and quality improvement, particularly in areas such as patient education, discharge planning, and navigation, but meaningful use depends on adequate time, facilitation, and integration with existing nursing quality-improvement processes. Taken together, these studies suggest that OHL frameworks and tools can act as levers for nursing-led improvement when they are embedded into routine audit, feedback, and quality-improvement cycles.

#### Conceptual area 3: enablers and barriers to OHL that shape nursing work

The potential of organizational enablers to support nurses' OHL work were discussed and described in three studies. Nurses' abilities to deliver HL-sensitive care were shaped by multilevel enablers and barriers.

Rathmann et al. (25) concluded that future OHL measures and initiatives should strengthen the development of, and access to, target-group-specific health information and create health-literate working and living environments, thereby functioning as enabling organizational contexts for staff and service users alike. Häberle et al. (22) reported associations between higher OHL and organizational characteristics including leadership commitment, interprofessional collaboration and integration with quality initiatives. Management mandates, internal coordinators, multidisciplinary assessment teams, workshops, and joint consensus meetings have also been discussed as key enablers of hospital OHL assessment (1).

Conversely, Zhu et al. (23) discussed organizational barriers to OHL development in some hospitals in China, including limited leadership commitment, resource and staffing constraints, inadequate staff training in HL, and rigid organizational structures, all of which directly constrained nurses' capacity to provide HL-sensitive care for older adults with complex needs. Stavdal et al. (26) similarly observed that limited attention to how nursing staff develop and maintain HL sensitivity represented an organizational barrier, with HL education often *ad hoc* and poorly integrated into ongoing professional development. In relation to HL education, Lubasch et al. (27) found nurses to be the most reached group in training, whilst physicians engaged least, illustrating how profession-specific dynamics may shape OHL implementation and nurse roles, and underscoring that nurses' OHL efforts can be amplified or constrained by organizational factors such as leadership commitment.

#### Conceptual area 4: implementation strategies and health literacy nurse champions

Across the included studies, implementation strategies to embed OHL typically combined staff education, use of OHL self-assessment tools, and iterative quality-improvement projects led or co-led by nurses. Stavdal et al. (26) illustrated how a structured HL competence programme for nurses, aligned with ward-level improvement goals, could increase nurses' confidence in communicating clearly and tailoring information, while also highlighting the importance of follow-up, supervision, and peer support to sustain practice change. Wu et al. (24) extended this by conceptualizing a “health-literate nursing system” in which designated HL nurse champions play a pivotal role in coordinating OHL activities, facilitating staff engagement with assessment tools, and linking frontline insights to managerial decision-making. Evidence supported the establishment of HL change agents, participatory co-design, repeated training, ward-level supporting materials, and nurses' suggestions for supervision and embedded reflection (27). Lubasch et al. (27) also found that HL training alone did not change working routines; change agents required stronger leadership backing. Stavdal et al. (26) illustrated the feasibility of a ward-based educational intervention to improve nurses' health literacy competencies. Such strategies, supported by leadership and embedded within existing governance structures, were seen as critical for maintaining momentum, integrating OHL into routine audits and accreditation processes, and ensuring that HL considerations are not viewed as an optional add-on but as a core element of quality and safety (26). Straßmayr et al. (1) viewed OHL implementation as an organizational self-assessment strategy, recommending a mandated, coordinator-led, multidisciplinary seven-step OHL-Hos process supported by introductory workshops, tailored completion by professional groups, and explicit assignment of responsibility for follow-up of OHL action plans. These implementation strategies underscore that nurses can act not only as communicators and educators at the bedside, but also as organizational change agents driving the adoption and normalization of OHL practices.

## Discussion

From a conceptual analysis perspective, recent work on OHL marks a big shift from viewing HL as an individual patient skill set to understanding it as a core responsibility of healthcare organizations, providing critical context for nursing practice (20). The illustrative literature considered in this conceptual analysis suggests that nurses may have a central role in operationalizing this organizational responsibility through communication, patient education, care navigation, advocacy, and participation in quality-improvement activities. Although communication and information practices were the most visible aspects of nurses' OHL work, the mini-review also underscores the importance of broader organizational conditions, including leadership and governance, workforce capability, resourcing and support systems. This indicates that, whilst OHL is recognized across multiple domains, it is often implemented in a fragmented manner, unless it is deliberately embedded within organizational structures and routine quality-improvement processes. One quality-improvement initiative supports this conclusion by showing that OHL-related goals become more actionable when these are integrated into existing improvement cycles, supported by dedicated project teams, iterative testing of changes, and regular feedback to frontline nursing staff (28).

Across the four pre-specified areas discussed in this conceptual analysis, the included studies provide examples of OHL frameworks and self-assessment tools, such as the International Self-Assessment Tool for Organizational Health Literacy of Hospitals (OHL-Hos), derived from the Vienna Concept of Health Literate Hospitals and Healthcare Organizations (V-HLO), are increasingly applied within hospital settings to guide interdisciplinary teams, including nursing staff, in systematically identifying gaps in communication, navigation and patient support. However, limited leadership commitment, resource constraints and insufficient staff training remain substantial barriers to effective implementation. OHL-Hos is now used in European hospitals to structure such assessments, yet recent work by Straßmayr et al. (1) suggests that future instruments will need to retain common core domains while becoming more streamlined, context-sensitive, conceptually robust and rigorously validated. Taken together, these frameworks offer nurses and nurse leaders practical levers to drive improvements in communication, navigation and patient support, particularly when, as illustrated by Isibel's (28) programme, assessment findings are explicitly linked to quality-improvement plans, governance structures and reporting mechanisms.

Studies of enablers and barriers nonetheless highlight how leadership commitment, resourcing and structured staff development either enable or constrain HL-responsive nursing care at the bedside and across care pathways. The difference between HL and OHL in the professional setting should therefore be noted. Health literacy refers to nurses' individual competencies, including clear communication and teach-back techniques, whereas OHL goes a step further by embedding these skills within supportive organizational structures, including leadership, staff training and enabling systems. This distinction is important because it suggests that HL, on its own, may be insufficient to achieve truly person-centered care within nursing practice. Complementary work on HL champions and social-network approaches further suggests that sustained progress will depend on preparing and supporting nurses and other healthcare professionals to act as organizational change agents, linking hospital wards, community-based services and policy arenas, and ensuring that OHL is integrated into quality and safety agendas, rather than treated as an optional add-on in the healthcare setting.

## Strengths and limitations

This paper presents a conceptual analysis informed by a focused mini-review, not a systematic review or comprehensive evidence synthesis. Although transparent search and selection procedures were used to identify recent empirical studies, the search was restricted to English-language publications; consequently, relevant evidence published in other languages may not have been identified, particularly from settings where OHL research may be reported in local languages. The focused search was limited to selected databases, a defined publication period, consequently, relevant studies may not have been identified. Accordingly, the themes presented are intended to support conceptual development and generate practice-relevant hypotheses, rather than provide definitive estimates of effectiveness or universal recommendations. Several framing and background references were authored by members of the research team members; however, none of the included empirical studies were conducted by the authors, which may have influenced the conceptual framing of the paper. In addition, the current evidence base predominantly reports OHL from a managerial or organizational perspective, with relatively limited attention to in-depth nursing outcomes, so the transferability and specificity of our conclusions for nursing practice, particularly in low-resource or highly diverse settings, should be interpreted with caution. Together, these limitations underscore the need for more robust, preferably longitudinal or interventional, studies that examine how nurses actually use OHL frameworks to embed HL into their routine clinical practice.

## Research gaps and future directions

Despite conceptual and practical advances, important gaps remain. First, relatively few rigorously designed studies have evaluated nurse-led OHL interventions across diverse healthcare contexts, particularly in low- and middle-income countries. Second, there is limited understanding of how organizational factors, such as leadership support, workload, and digital infrastructure, shape nurses' HL roles. Third, OHL competencies and HL roles are not yet systematically integrated into nursing curricula, regulatory standards, or professional career frameworks, limiting the visibility of, and support for, nurses' OHL work. We believe that future research should prioritize co-designing OHL implementation tools with nurses and service users, using participatory approaches to adapt existing frameworks to local cultural and resource contexts. Mixed-methods evaluations can examine the impact of nurse-led OHL initiatives on service users' understanding, self-management, clinical outcomes, service use, and equity, while exploring mechanisms through which nurse champions influence organizational routines and culture.

At the policy level, embedding OHL within accreditation standards for nursing education, and practice through quality and safety indicators, and performance frameworks would create stronger system-level incentives for organizations to prioritize health-literacy-responsive care. Explicitly recognizing OHL competencies within accreditation and regulatory, for example professional registration, standards would legitimize nurse-led OHL roles, support workforce capability development, and align professional expectations with health-equity goals. Incorporating OHL measures into funding and performance reporting frameworks, particularly in primary care and community health settings, could further encourage sustained organizational investment in leadership, communication systems, and navigation support. Collectively, these policy mechanisms would shift responsibility from individual clinicians to organizations, enabling nurses to lead and embed OHL as a core component of routine, high-quality, and equitable healthcare delivery.

## Implications for practice

This mini review highlights several practical implications for nursing practice at an organizational level. Nurses have a key role in meeting patients' HL needs, particularly via clear verbal and written communication, information provision and patient education. Given the complexity of healthcare systems, embedding OHL may help simplify health information materials and support care navigation. In addition, nurses may contribute to OHL through engaging in quality improvement initiatives, collaborating with and leading interdisciplinary teams, and using validated OHL assessment tools. Nonetheless, we should be aware of the importance of organizational support, including leadership commitment and structured training, as the effectiveness of OHL activities in the clinical setting depends on organizational conditions. Strengthening these organizational conditions may enhance nurses' capacity to deliver person centered care in a HL-sensitive manner.

## Conclusion

Organizational health literacy (OHL) may support the delivery of more accessible, safe, and equitable healthcare. This conceptual analysis, informed by illustrative recent literature, suggests that nurses may contribute to operationalizing OHL through communication, patient education, care navigation, advocacy, and participation in quality-improvement activities. However, the extent, effectiveness, and sustainability of nurse-led OHL initiatives require further evaluation across diverse healthcare settings. In particular, OHL frameworks, self-assessment tools, and health literacy nurse champions emerge as key conceptual levers for embedding health-literacy, sensitive communication, navigation, and environments into routine care, provided they are supported by leadership, sufficient resourcing, and integration into education, quality improvement, and policy structures. Strengthening organizational conditions for nursing engagement with OHL may help to improve the accessibility and responsiveness of care. However, robust implementation and outcome evaluations are needed before firm conclusions can be drawn regarding effects on equity, patient empowerment or health-related outcomes, to ensure that OHL becomes a practical reality for nurses rather than remaining a largely conceptual aspiration in the healthcare arena.
